# Factors Influencing the Popularity of a Health-Related Answer on a Chinese Question-and-Answer Website: Case Study

**DOI:** 10.2196/29885

**Published:** 2021-09-28

**Authors:** Jinhui Li, Han Zheng, Xu Duan

**Affiliations:** 1 School of Journalism and Communication Jinan University Guangzhou China; 2 National Media Experimental Teaching Demonstration Center Jinan University Guangzhou China; 3 Wee Kim Wee School of Communication and Information Nanyang Technological University Singapore Singapore

**Keywords:** answer-response behaviors, Zhihu, HPV vaccine information, content features, context features, contributor features

## Abstract

**Background:**

Social question-and-answer (Q&A) sites have become an important venue for individuals to obtain and share human papillomavirus (HPV) vaccine knowledge.

**Objective:**

This study aims to examine how different features of an HPV vaccine–related answer are associated with users’ response behaviors on social Q&A websites.

**Methods:**

A total of 2953 answers and 270 corresponding questions regarding the HPV vaccine were collected from a leading Chinese social Q&A platform, Zhihu. Three types of key features, including content, context, and contributor, were extracted and coded. Negative binomial regression models were used to examine their impact on the vote and comment count of an HPV vaccine–related answer.

**Results:**

The findings showed that both content length and vividness were positively related to the response behaviors of HPV vaccine–related answers. In addition, compared with answers under the question theme *benefits and risks*, answers under the question theme *vaccination experience* received fewer votes and answers under the theme *news opinions* received more votes but fewer comments. The effects of characteristics of contributors were also supported, suggesting that answers from a male contributor with more followers and no professional identity would attract more votes and comments from community members. The significant interaction effect between content and context features further showed that long and vivid answers about HPV vaccination experience were more likely to receive votes and comments of users than those about benefits and risks.

**Conclusions:**

The study provides a complete picture of the underlying mechanism behind response behaviors of users toward HPV vaccine–related answers on social Q&A websites. The results help health community organizers develop better strategies for building and maintaining a vibrant web-based community for communicating HPV vaccine knowledge.

## Introduction

### Background

With the prevalence of Web 2.0, social media has become a significant venue for individuals to exchange health-related information. Compared with traditional information sources (eg, television and newspapers), social media provides more customized information with higher efficiency for users to access. Health information–seeking behaviors and needs on social media have inspired a number of recent research streams [1,2], engendering crucial insights into the treatment of various health issues such as diabetes, HIV, chronic illnesses, and mental health. Among them, a significant number of studies have focused on information related to human papillomavirus (HPV) vaccines on social media [3-6]. HPV is a common sexually transmitted infection responsible for most cervical cancer cases [7]. The HPV vaccine has been recognized as an effective method to prevent cervical cancer, but its vaccination rate among young women remains low in developing regions, such as 1.1% in Asia and 1.2% in Africa [8]. In China, only 3.1% of college students have been vaccinated against HPV [9].

The literature has suggested that HPV vaccine–related information on social media plays an important role in shaping attitudes and behaviors of individuals toward HPV vaccination [10-12]. For example, both a survey study [13] and a content analysis [14] showed that information about HPV vaccine safety concerns might affect HPV vaccination acceptance and behaviors. Although these studies provide valuable insights on knowledge sharing of HPV vaccines on different social media platforms, few of them have investigated what makes an HPV vaccine answer popular in social question-and-answer (Q&A) sites.

Social Q&A websites are a type of social media that were originally designed for knowledge creation and sharing. It presents the content with a *Q&A* structure, where users ask and answer questions of one another about a wide range of topics [15]. Recent years have witnessed the increasing popularity for the public to seek and share health information on social Q&A platforms [16,17]. Unlike other social media platforms that are often centered on tight social relationships, social Q&A websites are typically based on loose social connections between community members who share a common interest. It is especially important to understand what makes an answer popular, as it would facilitate the maintenance of a vibrant web-based community on social Q&A platforms [18]. However, little research effort has been made to examine the popularity of predictors of health-related answers, not to mention the answers about the HPV vaccine topic. To address this research gap, this study aims to advance the existing literature on social engagement of users on social Q&A platforms by investigating how different characteristics of an HPV vaccine–related answer affect response behaviors of users (ie, voting and commenting) on Zhihu, a Chinese social Q&A platform. The findings of this study could benefit academia and practice by identifying and formulating well-grounded strategies to encourage user engagement in health knowledge discussions on social Q&A websites, which could facilitate the amplification of HPV vaccine information on social media and eventually enhance vaccine acceptance and vaccination behavior.

### Literature Review and Hypotheses

Behavioral engagement of users (eg, voting and commenting) on social Q&A platforms is a key driver of platform growth and its long-term success [18]. The quality of answers on these platforms is critical for users’ engagement with the content and the platform. In this study, we are particularly interested in exploring various features of health-related answers that can shape the actual behavioral engagement of users on social Q&A websites. Previous studies have used existing theoretical frameworks to understand potential features. For example, based on the heuristic-systematic model, Wang et al [19] developed a conceptual framework consisting of content and sender factors that drive cancer information diffusion on social media. Some social Q&A studies [20,21] further emphasized the question features that may influence the user response rate, such as the topic and the posting time of a question. Inspired by the abovementioned studies, this study proposed three main features of a health-related answer that may affect response behaviors of users, including content (answer-related features), context (question-related features), and contributor (user-related features).

### Content Features

In the context of web-based communities, textual content is the basic element for conveying information. Content length, which is defined in this study as the number of words that a contributor writes for an answer, is often considered as an important indicator for comprehension and helpfulness of textual content. A post with a longer length indicates a greater likelihood of involving detailed descriptions [22], increasing the perceived usefulness of the audience [23]. As such, an answer containing more words is more likely to satisfy the health knowledge needs of users in a social Q&A environment. For example, in an HPV vaccine–related web-based discussion forum, a longer answer might include more details, such as the risks and benefits of the HPV vaccine or vaccine uptake experience [24]. Thus, long answers could convey more relevant knowledge to readers and aid their understanding of HPV vaccination.

The content vividness of an answer is another important feature that facilitates information dissemination on social Q&A platforms. In this study, we define content vividness as the amount of sensory information (eg, image) provided in an answer. Fang et al [18] argued that content vividness is useful for attracting the attention of users by increasing information richness and visual richness. On social media platforms, the vividness of a post is commonly evoked by multimedia, such as pictures or videos. Yin et al [25] found that using multimedia to complement textual content can increase information credibility in sharing posts. Furthermore, Jiang and Benbasat [26] observed that content vividness enhances user enjoyment. Hence, content vividness can bring both functional and emotional benefits to readers in a social Q&A environment.

According to the social exchange theory [27,28], when people receive help and support from peers in a social network, they feel the need to reciprocate such help. Applying this theory to the social Q&A context, users might feel obligated to pay back the contributors who share knowledge in an answer, especially when they perceive that the answer successfully fulfills their information needs through a detailed description and some visual aids. Post replying is the most common repaying behavior because it can promote the recognition and reputation of contributors [18]. Many studies have demonstrated that health-related content on social media predicts user social engagement, such as sharing or commenting [4,24]. For example, a study by Rus and Cameron [29] indicated that diabetes-related messages with images receive higher rates of user engagement compared with those without images. Similarly, Fang et al [18] also found that post length and information vividness affect the number of reply posts in a web-based community. Taken together, we propose the following hypothesis:

Hypothesis 1: content features, including (a) content length and (b) content vividness, are positively associated with response behaviors toward HPV vaccine–related answers.

### Context Features

The core concept of social Q&A platforms is that users are allowed to express an information need in the form of a question and seek answers from fellow users in the community [30]. The themes of these questions vary from one to another. For example, Sharon et al [31] found that vaccine-related questions focus on different topics, such as the benefits and risks of vaccination and vaccine schedules. These questions have become an essential part of a diverse and vibrant whole on many platforms, reflecting and supporting users with different goals and expectations [32]. Considering the variety of question themes, the way in which knowledge contributors answer a question might be different [33]. There are several early attempts to investigate user preferences for answering certain themes of questions. For instance, Hsieh and Counts [34] found that *socially conducive* questions are more favored by users, whereas Harper et al [35] indicated that *advice-seeking* questions receive longer answers than *fact-seeking* questions. Depending on the theme of the question, the context of answering that question is also different. Therefore, in this study, we argue that the question theme might serve as the context feature of an answer on a social Q&A platform.

Despite a growing body of literature investigating users’ response behaviors in web-based communities [18,29], limited research has examined how the situational context behind a question affects the popularity of its answers. In a social Q&A context, answers received from fellow users are often expected to address what the asker articulates in the question. In other words, users’ evaluation of these answers is closely related to the asker’s perceived expectations indicated by the questions [32]. In a study of the social Q&A site *Yahoo! Answers*, Kim et al [36] found that question themes shape the evaluation criteria of users to select the *best answer*. As an answer with a high evaluation score may trigger more social engagement (eg, liking and commenting) [18,37] and different question themes may be evaluated differently by the users, it is reasonable to assume that question themes can affect the number of likes and replies in an answer when people contribute HPV vaccine knowledge on a social Q&A platform. Therefore, we hypothesize the following:

Hypothesis 2: the question theme is associated with response behaviors toward HPV vaccine–related answers.

In addition to the main effects mentioned earlier, there might be an interaction effect between content and context features on response behaviors of users. It is possible that the effects of a long and vivid answer on response behaviors would be amplified within specific question themes. For instance, a more detailed answer with additional images to the question asking people’s experience of HPV vaccination might appeal to more users, thus triggering more discussion from the community members when compared with those questions under another theme (such as costs of HPV vaccines). In other words, more vivid, comprehensive answers under certain question themes might better satisfy health information needs of users and generate more user engagement. Understanding this interaction effect is valuable in addressing the practical needs of community developers who want to maximize user engagement on their social Q&A websites. Considering that there is limited research examining such synergistic effects on social engagement, we propose the following research question:

Research question: does the question theme moderate the effects of content length and content vividness on response behaviors toward HPV vaccine–related answers?

### Contributor Features

When browsing user-generated content on social media, users often evaluate the characteristics of a source to determine its credibility, which further affects their behavioral intentions. Source credibility theory [38] identifies source expertise and trustworthiness as key elements that affect information credibility. Whether a message sender is perceived as highly credible is determined by others’ evaluation of their knowledge, occupation, social training, or experience [39]. In the web-based environment, such evaluations are based on the social cues provided by the contributor in the site network, such as gender, education level, profession, and the number of followers. These cues may serve as heuristics that impact source credibility, translating to the contributor’s perceived expertise and trustworthiness.

For instance, compared with female sources, information coming from male sources is more likely to be perceived as credible, especially on information-focused topics [40]. Furthermore, those who have higher education levels are perceived to have sufficient expertise; thus, they are considered credible [41]. Profession also contributes to the perceived expertise of the contributor, which predicts perceived credibility [42,43]. Moreover, users who have more followers are more likely to be considered trustworthy than those with a low number of followers [44]. All these social cues of source credibility will eventually affect other social engagement of users with the information. Finally, Kareklas et al [45] conducted a web-based experiment among participants who viewed 4 fictitious individuals who either expressed pro- or antivaccination viewpoints. Their results suggest that individuals who are perceived as credible are more likely to shape consumers’ vaccination attitudes and behavioral intentions. As such, users of social Q&A platforms constantly check the credibility of an answer’s contributor through social cues (ie, gender, education level, follower account, and profession); such credibility evaluations are critical in affecting users’ participation in blog-mediated communication [46]. Thus, we propose the following hypothesis:

Hypothesis 3: contributor features such as (1) gender, (2) education level, (3) follower count, and (4) profession are associated with response behaviors toward HPV vaccine–related answers.

## Methods

### Data Collection

**Figure 1:**
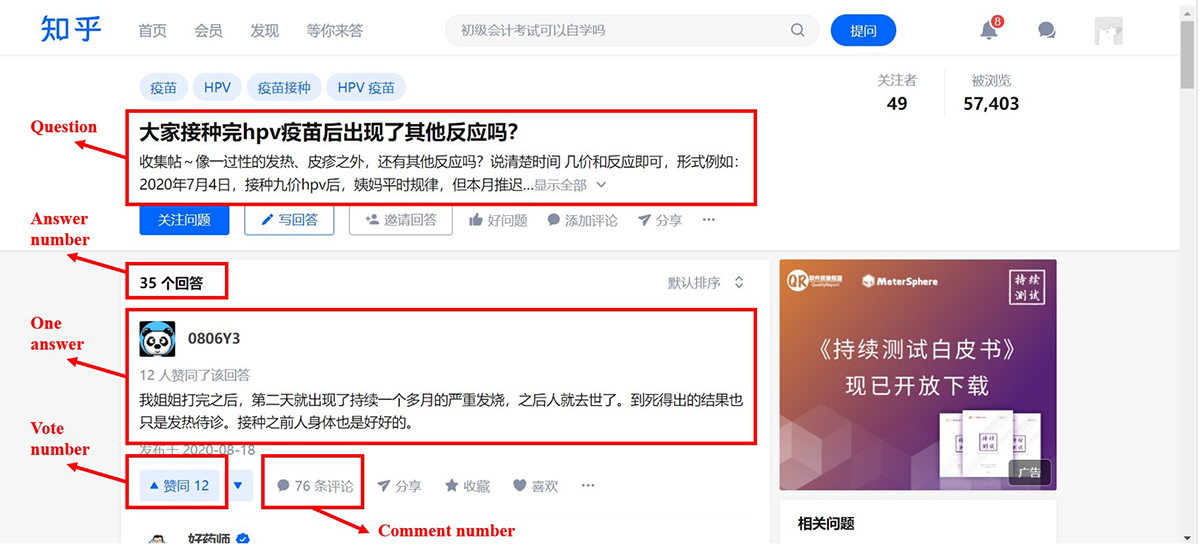
Screenshot of the main interface of Zhihu.

Data were collected from a leading social Q&A website in Zhihu, China [1]. Launched in 2011, Zhihu is the largest Chinese social Q&A website with over 160 million registered users and 26 million daily active users as of July 2018 [47]. It is a platform that facilitates knowledge creation and sharing in the public. In Zhihu, questions are created, answered, edited, and managed by users. There is a wide range of topics discussed on the Zhihu platform, enabling users to have convenient access to specific questions and answers. For example, on the platform, HPV vaccine (Chinese “宫颈癌疫苗”) is a unifying topic where one can find relevant questions and answers. A screenshot of the Zhihu interface is shown in Figure 1, which shows one question related to the HPV vaccine and the answers contributed by the community members. Under this particular topic, users could raise HPV vaccine–related questions while other community members with relevant knowledge (ie, contributors) answer the questions. We extracted all the questions with respective answers relating to the HPV vaccine available on Zhihu using the Python Web Crawler (Python Software Foundation) in March 2019. A total of 2953 answers with 270 corresponding questions were collected for the study. In addition, profile information of contributors (eg, gender and education) was also collected for data analysis.

### Operationalization of Study Variables

We hypothesized that three types of features (ie, content, context, and contributor features) are determinants of response behaviors on social Q&A platforms. The concepts and measurements used in this study are summarized in Table 1. First, content features included content length and content vividness, which were operationalized, respectively, as word count (WC) and image count (IC) in a specific answer. Second, context features were assessed using the theme of each answer. In total, 3 research assistants who majored in communication were recruited to independently review all 270 questions and code them into different categories. They further discussed, based on the initial subject of the questions, and resolved the discrepancies in the coding results until a consensus was reached. To this end, a coding scheme including three types of question themes was developed: (1) benefits and risks, (2) vaccination experience, and (3) news opinions. Detailed descriptions and examples are provided in Table 1. Third, contributor features were measured by four types of personal information of each user on Zhihu, including gender (male or female), education (presented or not presented), profession (presented or not presented), and follower count. Finally, as the dependent variable of this study, response behaviors were assessed using two metrics of any given answer on Zhihu: (1) vote count (ie, the number of users who show *agreement* with one answer) and (2) comment count (ie, the number of comments in one answer).

**Table 1:** Measurements of constructs.

Construct and description					Value
**Content features**					
	**Word count, mean (SD)**				
		The number of words in answer i		307.39 (1088.77)	
	**Image count, mean (SD)**				
		The number of images in answer i		0.42 (1.98)	
**Context features**					
	**Question theme, n (%)**				
		Theme 1. Benefits and risks: referring to questions about positive and negative (side effects) effects of HPV^a^ vaccine, for example “网传HPV疫苗将导致不孕及其他副作用是真的吗？ (Is the web-based information true that the HPV vaccine will cause infertility and other side effects)”		542 (18.43)	
		Theme 2. Vaccination experience: referring to questions about procedure and cost of HPV vaccine, for example “深圳或香港怎么预约HPV疫苗 (How to make an appointment for HPV vaccine in Shenzhen or Hong Kong)”		1917 (65.18)	
		Theme 3. News opinions: referring to questions about opinions or discussion of public news relating to HPV vaccine, for example “如何看待时隔11年后宫颈癌二价疫苗终于在中国大陆上市 (How do you think that the Cervarix vaccine has finally been launched in mainland China after 11 years)”		482 (16.39)	
**Contributor features**					
	**Gender, n (%)**				
		**The author’s gender of answer i**			
			Male	1919 (65.25)	
			Female	1022 (34.75)	
	**Education, n (%)**				
		**If the author of answer i reveals educational background**			
			Presented	673 (22.88)	
			Not presented	2268 (77.12)	
	**Profession, n (%)**				
		**If the author of answer i reveals professional background**			
			Presented	443 (15.06)	
			Not presented	2498 (84.94)	
	**Follower count, mean (SD)**				
		The number of fans of answer i’s author at time t		2145.98 (27,095.82)	
**Response quantity**					
	**Vote count, mean (SD)**				
		The number of votes of answer i at time t		10.16 (137.42)	
	**Comment count, mean (SD)**				
		The number of comments of answer i at time t		4.44 (33.56)	

^a^HPV: human papillomavirus.

### Data Analysis

**Figure 2:**
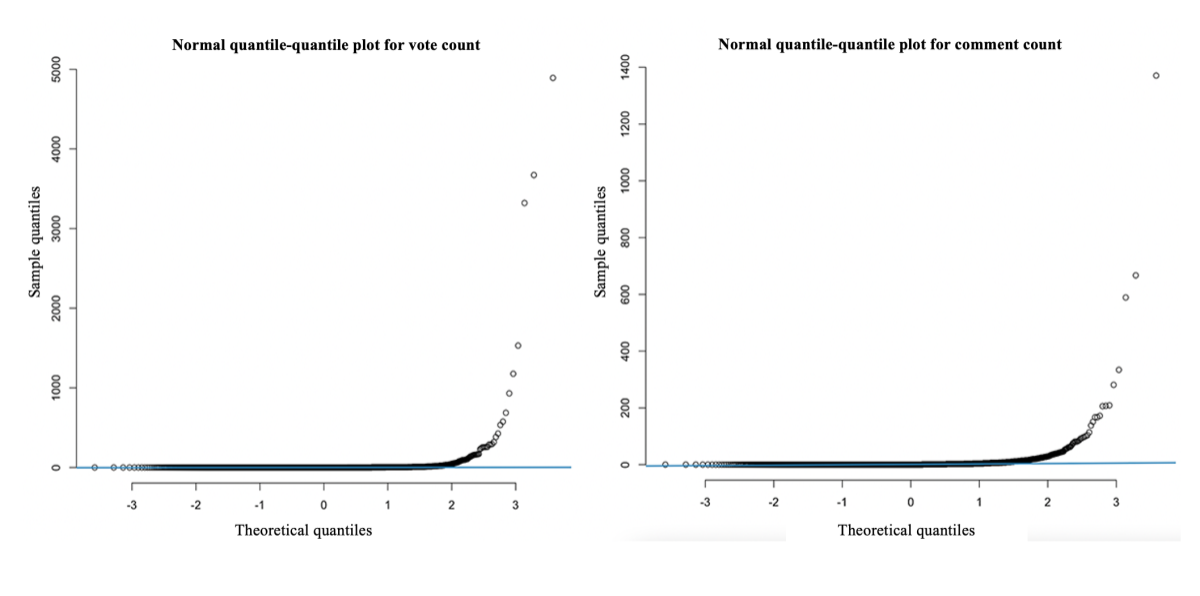
Quantile-quantile plots for vote count and comment count.

The two measures of response behaviors (ie, vote count and comment count) were count data, a type of data in which the observations can take only the nonnegative integers. As shown in Table 1, the variances of vote count and comment count (137.42 and 33.56, respectively) were significantly greater than their conditional means (10.16 and 4.44, respectively), indicating that the data distributions for the two variables were overdispersed. Furthermore, the quantile-quantile plots also show that vote count and comment count did not follow a normal distribution, and there were numerous extreme values (Figure 2). In this case, regression models using ordinary least squares might produce inconsistent, biased results [48]. Therefore, we used negative binomial regression modeling as it does not assume an equal mean and variance and introduces a parameter to correct for overdispersion.

We established hierarchical regression models to examine the influence of different factors on vote and comment counts. Model 1 included measures of content, context, and contributor features to test the main effects. Following this, the interactions between the measures of content and context features were added in model 2. In addition, our model included a variety of independent variables in one setting, and Bonferroni correction was used to avoid multiple comparisons fallacy. More specifically, the Bonferroni-corrected *P* value is defined as *α/k (number of tests)* [49,50]. Accordingly, we applied a significance level of *P*=.02 (*P*=.05 is divided by three themes) in our analysis. Finally, we performed the variance inflation factor test to check for multicollinearity, and the results indicated that the variance inflation factors for study variables ranged from 1.03 to 1.95, which were all below the recommended value of 10 [51]. Thus, there is no multicollinearity in the proposed empirical models.

## Results

### Main Effects

Tables 2 and 3 show the effects of various factors on vote and comment counts. Hypotheses 1(a) and 1(b) show that content length and content vividness are positively associated with response behaviors of users. As is evident from model 2 in the two tables, the WC of an answer was positively associated with both vote count (β=7.96E-04; incidence rate ratio [IRR]=1; *P*<.001) and comment count (β=1.35E-04; IRR=1; *P*=.004). This finding supports hypothesis 1(a). The IRRs or exponentiated values were calculated to interpret the regression coefficients of the indicator variables. For example, a one-unit change in WC increases the answer’s rate of obtaining a vote and a comment by a factor of 1, while holding all other predictors constant in the model. Similarly, the IC in an answer was positively related to both vote count (β=3.96E-01; IRR=1.49; *P*<.001) and comment count (β=2.19E-01; IRR=1.25; *P*=.02). Thus, hypothesis 1(b) was supported.

Hypothesis 2 stated that the context feature of an answer (ie, a question theme) would affect response behaviors. We found that compared with the reference group *benefits and risks* (theme 1), *news opinions* (theme 3) was positively related to vote count (β=4.76E-01; IRR=1.61; *P*=.007), whereas *vaccination experience* (theme 2) was not significantly related to vote count (β=−2.87E-01; IRR=0.75; *P*=.04). In addition, compared with the reference group *benefits and risks*, *news opinions* had a negative effect on comment count (β=−4.35E-01; IRR=0.65; *P*=.004).

As for hypothesis 3, we stated that various contributor features would induce an HPV vaccine–related answer’s response behavior. The results showed that male gender (β=6.68E-01; IRR=1.95; *P*<.001) and follower count (β=2.98E-01; IRR=1.35; *P*<.001) were positively associated with vote count, whereas presence of profession (*P*=.03) and education (*P*=.90) was not significantly associated with vote count. Similarly, male gender (β=2.21E-01; IRR=1.25; *P*=.01) and follower count (β=1.70E-01; IRR=1.19; *P*<.001) had similar positive effects on comment count, whereas presence of profession (*P*=.03) and education (*P*=.64) had no impact on comment count.

**Table 2:** Results of negative binomial regression model (vote count).

Variables			Model 1						Model 2				
			Estimate (SE)		*P* value		IRR^a^		Estimate (SE)		*P* value		IRR
**Content features**													
	Word count	1.42E-03 (4.56E-05)		<.001		1.00		7.96E-04 (5.42E-05)		<.001		1.00	
	Image count	8.43E-01 (2.52E-02)		<.001		2.34		3.96E-01 (1.08E-01^)^		<.001		1.49	
**Context features (question theme)**													
	Theme 1^b^ (reference)	—^c^		—		—		—		—		—	
	Theme 2^d^	2.89E-02 (1.34E-01)		.83		1.04		−2.87E-01 (1.38E-01)		.04		0.75	
	Theme 3^e^	5.39E-01 (1.70E-01)		.002		1.81		4.76E-01 (1.76E-01)		.007		1.61	
**Contributor features**													
	Gender (male)	6.33E-01 (1.06E-01)		<.001		1.97		6.68E-01 (1.05E-01)		<.001		1.95	
	Education (presented)	6.46E-02 (1.49E-01)		.67		1.10		4.71E-02 (1.48E-01)		.90		1.05	
	Profession (presented)	−3.75E-01 (1.69E-01)		.03		0.65		−3.57E-01 (1.67E-01)		.03		0.70	
	Ln(fans count)	3.07E-01 (2.74E-02)		<.001		1.31		2.98E-01 (2.73E-02)		<.001		1.35	
**Interaction**													
	WC^f^ Theme 1^b^ (reference)	—		—		—		—		—		—	
	WC Theme 2^d^	—		—		—		8.03E-04 (1.04E-04)		<.001		1.01	
	WC Theme 3^e^	—		—		—		1.98E-04 (2.06E-04)		.34		1.00	
	IC^g^ Theme 1^b^ (reference)	—		—		—		—		—		—	
	IC Theme 2^d^	—		—		—		4.88E-01 (1.11E-01)		<.001		1.63	
	IC Theme 3^e^	—		—		—		−4.69E-01 (1.95E-01)		.02		0.63	
**Model fit**													
	Null deviance (df^h^)	3327.6 (2940)		—		—		3397.7 (2940)		—		—	
	Residual deviance (df)	2051.1 (2931)		—		—		2054.4 (2927)		—		—	
	AIC^i^	9433.7		—		—		9406.6		—		—	

^a^IRR: incidence rate ratio.

^b^Theme 1: benefits and risks.

^c^Not available.

^d^Theme 2: vaccination experience.

^e^Theme 3: news opinions.

^f^WC: word count.

^g^IC: image count.

^h^df: degree of freedom.

^i^AIC: Akaike information criterion.

**Table 3:** Results of negative binomial regression model (comment count).

Variables			Model 1						Model 2				
			Estimate (SE)		*P* value		IRR^a^		Estimate (SE)		*P* value		IRR
**Content features**													
	Word count	9.41E-04 (3.88E-05)		<.001		1.00		1.35E-04 (4.64E-05)		.004		1.00	
	Image count	1.96E-01 (2.14E-02)		<.001		1.22		2.19E-01 (9.26E-02)		.02		1.25	
**Context features (question theme)**													
	Theme 1 (reference)	—^b^		—		—		—		—		—	
	Theme 2	9.662E-02 (1.119E-01)		.38		1.10		−1.36E-01 (1.15E-01)		.24		0.87	
	Theme 3	−2.370E-01 (1.450E-01)		.10		0.79		−4.35E-01 (1.51E-01)		.004		0.65	
**Contributor features**													
	Gender (male)	1.90E-01 (9.01E-02)		.04		1.21		2.21E-01 (8.97E-02)		.01		1.25	
	Education (presented)	−1.05E-01 (1.26E-01)		.40		0.90		−1.04E-01 (1.26E-01)		.64		0.90	
	Profession (presented)	−2.98E-01 (1.43E-01)		.04		0.74		−3.04E-01 (1.43E-01)		.03		0.74	
	Ln(fans count)	1.67E-01 (2.31E-02)		<.001		1.18		1.70E-01 (2.32E-02)		<.001		1.19	
**Interaction**													
	WC^c^ Theme 1^d^ (reference)	—		—		—		—		—		—	
	WC Theme 2^e^	—		—		—		9.02E-04 (8.95E-05)		<.001		1.00	
	WC Theme 3^f^	—		—		—		1.01E-03 (1.77E-04)		<.001		1.00	
	IC^g^ Theme 1 (reference)	—		—		—		—		—		—	
	IC Theme 2	—		—		—		−1.11E-02 (9.57E-02)		.91		0.99	
	IC Theme 3	—		—		—		−4.26E-01 (1.72E-01)		.01		0.65	
**Model fit**													
	Null deviance (df^h^)	2996.0 (2940)		—		—		3025.0 (2940)		—		—	
	Residual deviance (df)	2398.4 (2931)		—		—		2401.1 (2927)		—		—	
	AIC^i^	10,711		—		—		10,700		—		—	

^a^IRR: incidence rate ratio.

^b^Not available.

^c^WC: word count.

^d^Theme 1: benefits and risks.

^e^Theme 2: vaccination experience.

^f^Theme 3: news opinions.

^g^IC: image count.

^h^df: degree of freedom.

^i^AIC: Akaike information criterion.

### Interaction Effects

Finally, the research question asked whether there was an interaction effect between the content and context features on response behaviors. First, for the moderating effect of WC and question theme, compared with the reference group *WC Theme 1: benefits and risks*, the interaction *WC Theme 2: vaccination experience* was positively related to vote count (β=8.03E-04; IRR=1.01; *P*<.001) and comment count (β=9.02E-04; IRR=1; *P*<.001). Moreover, the interaction between WC and news opinion was only positively associated with comment count (β=1.01E-03; IRR=1; *P*<.001). Second, for the moderating effect of IC and question theme, compared with the reference group *IC Theme 1: benefits and risks*, the interaction *IC Theme 2: vaccination experience* was positively associated with vote count (β=4.88E-01; IRR=1.63; *P*<.001). In contrast, the interaction *IC Theme 3: news opinions* was negatively associated with vote count (β=-4.69E-01; IRR=0.63; *P*=.02) and comment count (β=-4.26E-01; IRR=0.65; *P*=.01).

## Discussion

### Principal Findings

On social Q&A websites, social engagement of users toward an answer creates complex social feedback effects, which influence the knowledge-sharing behavior of a contributor in the future. Through the lens of existing theories, including social exchange theory and source credibility theory, we identified seven essential features from three distinct dimensions (ie, content, context, and contributor features) that may be associated with the heterogeneity in the answer-response behaviors, and evaluated the impacts of these features in a holistic and comprehensive research model that has not yet been investigated in the extant literature. The empirical results of this study lead to important findings in health communication and social media research. Overall, the results suggest that the content, context, and contributor features of an HPV vaccine–related answer are associated with other users’ engagement on a social Q&A site. At the content level, the findings show that both post length and image number positively impact the number of votes and comments toward a certain answer. Although similar results have been found in user-generated content on social media or general web-based communities [18,52,53], this study further extends these key conclusions to the context of communicating health information on emerging social Q&A websites. Specifically, our findings highlight that richness and vividness of an HPV vaccine–related answer can increase the willingness of other members to respond to this post. The findings also support the applicability of social exchange theory to understand the effect of content features on the response behaviors of users. From this theoretical perspective, people tend to show support and gratitude to those who spend time and effort contributing to comprehensive knowledge [54]. Votes and comments are thus regarded as reciprocal behavior from the users who have successfully had their information and psychological needs addressed by the rich and vivid answers. As the awareness of people of an HPV vaccine has been reported to be low in China [55], this study highlights the need to include helpful and concrete messages with appropriate visual illustrations to promote relevant health knowledge sharing in the social Q&A context.

At the context level, the results reveal that the question theme can affect the response behaviors of users toward a corresponding answer on social Q&A websites. Several existing studies in the health promotion research domain [56,57] have shown that posts under certain health topics are more likely to trigger information sharing of users. This study contributes to this body of literature by suggesting that answers responding to the topics about benefits and risks of HPV vaccine have received fewer votes but more comments from web-based members, compared with those discussing relevant news of HPV vaccine. One possible explanation for this interesting observation is that, due to relatively low awareness about HPV vaccination in China, it may be difficult for the users to vote for an answer when they perceive the effects and safety of HPV vaccination as controversial. Instead, by having more latitude for self-expression than a simple vote, they are more likely to share their opinions regarding these controversial topics, thus generating more comments.

As for the characteristics of contributors, gender has a relatively large effect on response behaviors (both vote and comment count), followed by follower counts. Drawing support from source credibility theory, this study confirms the role of the demographic characteristics of a contributor as important credibility indicators in web-based knowledge-sharing communities. The findings have emphasized how these key accessible cues of source credibility of a contributor drive participation of other members in their answers on social Q&A websites. In general, an HPV vaccine–related answer posted by a male contributor with more followers but without professional identity would attract more users’ engagement from community members. Gender is widely recognized as an important demographic factor in studies of credibility perceptions [58,59] and web-based behaviors [60,61]. A study conducted on Facebook [62] found that compared with women users who tend to share more personal issues, men discuss more general public topics, which ultimately elicit more comments. Consistent with the literature, our study shows that even for some *female-related* public topics such as HPV vaccine, a contributor’s gender has the same influence on the response behaviors. Furthermore, contributors who have more followers would trigger more votes and comments regarding their posted answers. Compared with newcomers, they know how to create content that attracts attention, recognition, and favorable feedback from other users and are motivated to maintain their reputation in the web-based community [18]. However, this study did not support the notion that the presence of professional and educational profiles in an answer would affect its votes and comments. This conclusion is inconsistent with prior studies, which demonstrated that individuals often perceive opinions of experts as trustworthy and therefore are more likely to agree with them [63,64]. A possible explanation might be that nearly half of the Zhihu answer contributors who revealed their professional information in our data set are from the health insurance or commercial health industry. Users may not consider their posts as objective as actual health experts (such as doctors in public hospitals) because of the potential commercial interests of HPV vaccines.

More importantly, this study provides novel evidence related to the interaction effects between content and context features on response behaviors. In particular, long and vivid answers about HPV vaccination experience are more likely to receive users’ votes and comments than those about risks and benefits. This finding suggests that community members prefer to read and discuss more comprehensive answers about personal vaccination stories rather than medical facts about vaccines [24]. Such stories might provide users with more meaningful and customized guidance on how to take up an HPV vaccine in terms of its costs, schedule, and feelings, especially for those who intend to do so. Interestingly, we found that when answering a question about vaccine news, answers with more images received fewer votes and comments. It is possible that users might expect answers that unpack the systemic causes and consequences of public news related to HPV vaccines, for example, the reasons and results of the delays in approving HPV vaccines in mainland China. When it comes to HPV news discussion, users show a more favorable attitude toward answers using thematic framing instead of episodic framing [65]. Adding numerous images in this scenario might deteriorate the scientific value of such answers.

### Limitations and Future Directions

This study has several limitations that may open directions for future research. First, because of the nature of a content analysis, this study only focuses on observed answer-response behaviors in social Q&A platforms, failing to explore the psychological processes underlying these behaviors. Although there is existing literature that discusses the cognitive mechanisms of how social media information shapes decision-making behaviors [66], future studies should use surveys or experimental methods to further empirically investigate the underpinning process of response behaviors in this particular health context. Second, the content features measured in this study are relatively abstract and broad, which might not be able to capture the dynamics of the concepts. It is worthwhile for future studies to explore more detailed content features, such as readability, narrativity, and coherence, which could inform public health professionals to take more specific and effective actions to promote user engagement on social Q&A websites. Third, as the primary aim of this study is to investigate web-based health answers on a Chinese social Q&A website, the findings may not be generalizable to social Q&A websites in other cultural contexts. As a result, more studies need to be conducted within different cultural contexts to further validate our findings and provide additional insights into the potential cultural influence on HPV vaccine answer-response behaviors. Finally, the wide spread of misinformation on social media is noteworthy. Although beyond the scope of this study, one future direction is to consider the veracity of information regarding HPV vaccination shared on social Q&A websites, for example, using a data mining method to examine the relationship between the truthfulness of an answer and its engagement metrics (ie, number of votes and comments).

### Research and Practical Implications

This study contributes to the existing body of literature research in several ways. First, to our knowledge, this is one of the first attempts to investigate the factors predicting users’ response behaviors toward health-related answers on social Q&A platforms, under a novel and comprehensive framework with features from three different layers: content, context, and contributor. The findings pave the way for understanding the social dynamics of emerging web-based knowledge-sharing communities. The framework established in this study has the potential to inspire future research to extend its applicability to other health issues on social media platforms. Second, the findings emphasize the critical role of the question theme in facilitating social engagement in the web-based knowledge-sharing context. It contributes to a complete account of contextual effects within social Q&A platforms, seldom covered in previous research. Finally, the study extends the current web-based information behavior literature by examining the interaction effect between content and context features on social Q&A websites. Although many studies have highlighted the need for comprehensive content in web-based communities [67], they neglected the confluence of comprehensive contents and contextual topics that collectively induce response behaviors. This study provides a clearer picture of why people respond to health-related information on social Q&A websites by considering the synergistic effects between content and question themes.

From a practical perspective, the results can also help health practitioners and knowledge contributors on social media to better understand the information needs and preferences of the public regarding HPV vaccines. By identifying the key factors and their interaction effects that drive users’ response behaviors, this study helps health professionals develop better strategies for building and maintaining a vibrant web-based community for sharing HPV knowledge. For instance, health care providers and health communicators may use social Q&A platforms to develop campaigns to promote public awareness of HPV vaccination, especially to leverage the influence of opinion leaders on the platforms, as shown in the findings regarding contributors in this study. Specifically, the findings on characteristics of a contributor imply that male contributors are advisable to be more involved in the discussions of topics that are traditionally considered *female-only* topics, such as HPV vaccination. Perhaps, instead of framing it as a *woman’s issue*, practitioners might consider involving more men as HPV vaccine spokespersons, educating the public that HPV infection is not a woman’s issue but one that challenges both men and women as the disease can be transmitted across genders [68]. Health behaviors of both genders play a crucial role in the health of both men and women. As such, by engaging more men in cervical cancer prevention campaigns, practitioners are better able to erase the discrimination of *females-only issues*, increase HPV vaccination rate, and work toward a wider, healthier community in which both men and women are a part of.

In addition, social Q&A platform managers and developers can enact relevant policies and/or adopt useful tools to encourage comprehensive health content with high-quality images, such as applying optimal image-uploading technologies or offering extra incentives to informative answers. Furthermore, as implied in our findings, important members with a large group of followers (eg, opinion leaders or experts) on social media play a crucial role in engaging a wider public to discuss HPV vaccines. Therefore, social Q&A platform managers may incentivize knowledge contributors at the platform level to promote comprehensive, high-quality health content. For instance, they can apply certain algorithms to award informative answers with credits or privileges to encourage them to consistently share validated and useful information about HPV vaccines. General answer contributors should adapt different content strategies for different question topics to attract attention and feedback from other users. For instance, inserting more pictures in an answer is useful for questions about personal stories and experience, but it is not advised for questions about HPV news discussion.

## Abbreviations

HPV: human papillomavirus
IC: image count
IRR: incidence rate ratio
Q&A: question-and-answer
WC: word count
